# Establishment of the microstructure of porous materials and its relationship with effective mechanical properties

**DOI:** 10.1038/s41598-023-43439-6

**Published:** 2023-10-23

**Authors:** Kangni Chen, Hongling Qin, Zhiying Ren

**Affiliations:** Department of Vehicle Engineering, Institute of Metal Rubber & Vibration Noise, Fuzhou University, Fuzhou, 350116 China

**Keywords:** Mechanical engineering, Materials for devices

## Abstract

In this study, a porous structure for a porous liquid storage medium is generated, and the homogenization theory based on displacement boundary conditions is used to predict the effective mechanical properties. The relationship between the porous material’s macroscopic mechanical properties and microstructure is next analyzed. In order to establish the relationship between the microstructure of porous materials and their macroscopic mechanical properties, assuming that the pores grow along the *z* direction, a method is proposed to generate 3D open-cell porous materials based on six design parameters (i.e., the number of pores, porosity, irregularity of pore distribution, the randomness of pore growth in the *x* and *y* directions, and randomness of pore size). Since the porosity of oil-bearing materials ranges from 20 to 30%, the porosity of the RVE (Representative Volume Element) was kept under control at about 25%, and the effect of the six design factors on the mechanical properties of the RVE was investigated. Utilizing SLA 3D printing technology, specimens were produced, and compression tests were used to show how useful the results of the numerical analysis were. The results demonstrated that after the number of RVE pores reaches 9, the numerical results have good repeatability. The irregularity of the initial pore distribution has little effect on the effective mechanical properties of the RVE. At the same time, the increase in the randomness of pore growth and the randomness of pore size increases the degree of weakening of the mechanical properties in the z-direction, while reducing the degree of weakening in the x and y directions, but the latter has a smaller impact. Furthermore, there is a superimposition effect of design parameters on the RVE.

## Introduction

The porous fluid storage medium is a solid–liquid biphasic complex inspired by biological articular cartilage, which can self-compensate for lubrication by precipitating fluid through the pores under the synergistic effects of external loads, frictional heat and siphoning. It is widely employed in the domains of oil-bearings, porous bionic bones, and self-lubricating ball linear guides due to its low manufacturing cost and self-circulating lubrication properties^1^. The solid phase of porous fluid storage media is typically made using techniques like cold pressing, hot sintering, and 3D printing^2^, and the part is also known as porous material. Studies have shown that increasing the porosity of porous materials improves their ability to store fluid, which improves their lubrication performance^3^, but it weakens the mechanical properties of the material, such as compressive strength^4–6^. As the requirements for equipment service life and reliability increase, oil-bearings need to optimize the internal pore microstructure to balance the contradiction between their load bearing and lubrication performance^7^, and self-lubricating ball linear guides need to control the direction of pore openings to improve lubrication performance and lengthen service life^8^. Therefore, the solution lies in determining the mapping relationship between the pore microstructure and its macro mechanical properties and designing the microstructure of porous materials based on the service conditions of porous fluid storage media.

This line of research has its roots in the investigation of the mechanical properties of typical porous structures found in nature. Typically, the elastic strut network model created by Gent and Thoma in their investigation of the elastic deformation of foams^9^, the orthogonal cube constitutive model developed by Gibson and Ashby^10^. Later, Gibson and Ashby also developed the model's elastic bending deformation and plastic yielding mechanisms based on the hexagonal honeycomb structure's elastic modulus and Poisson's ratio under two mutually perpendicular loads. For open-cell aluminum foam materials, other researchers have developed a constitutive model and proposed a tetrakaidecahedral model^11–13^. These models are better suited to determining how the mechanical properties of uniformly regular thin-walled porous materials with high porosity relate to their microstructure. The effective mechanical properties of porous materials can also be calculated using a variety of numerical computational methods^14–16^. Typical examples include the Mori–Tanaka model (M–T) for calculating the effective mechanical properties of a material based on the pore slenderness ratio^17^ and the Three-Phase Model (TPM) for calculating the effective shear modulus of a material based on porosity^18^. All these numerical models take into account the irregularity of porous materials to some extent, but most studies only characterize the pore characteristics in terms of porosity and the constants associated with the pore shape. The relationship between the microstructural features of porous materials and their macroscopic mechanical properties cannot be adequately described by these parameters. The microstructural can, however, be accurately represented by the finite element method (FEM), which is based on a model structure.

Finite element analysis based on RVE (Representative volume element) is an effective method for predicting the effective mechanical properties of porous materials. And among them, how to generate the RVE of porous materials becomes the key to the problem. Due to its ability to generate random and irregular polygons, the Voronoi diagram method is frequently used to generate random geometry models of porous materials. This method was proposed by Silva et al.^19^ to generate a 2D Voronoi random model with uniform wall thickness using pore shape irregularity and porosity as design parameters, and discovered that the mechanical properties of high porosity porous materials were less dependent on pore shape irregularity. They then looked into the model more thoroughly and discovered that the removal of some cell walls, which had little impact on porosity, resulted in a sharp decrease in the material's useful mechanical properties^20^. Chen et al.^21^ considered six cells (pore particle) random defects based on the 2D Voronoi model, namely cell size variation, cell wall fracture, cell wall misalignment and cell absence, and found that cell edge fracture had the greatest effect on the yield strength of 2D foam materials. The random geometry models used in the above studies are based on the stochastic nature of one type of porous material (e.g., irregularities in pore shape and wall thickness). The microstructure of a porous material typically involves two or more random elements. Li et al.^22^ discovered that the effective elastic modulus of 2D foams was affected by the irregularity of pore shape and wall thickness, and that this effect increased as the porosity decreased. They did this by using porosity, cell shape irregularity, and wall thickness inhomogeneity as design parameters based on the Voronoi diagram method. Guo et al., investigated the degree of anisotropy of 2D porous materials based on the 2D Voronoi model and the 2D randomly distributed circular pore model, using porosity and pore number as design parameters. According to the findings, 2D porous materials’ degree of anisotropy decreases as the number of pores increases and increases as the porosity increases^5^. However, it is obvious that 2D RVE is insufficient to adequately describe the intricate microstructure of porous materials. As a result, it is still necessary to develop a 3D RVE that considers the microscopic random characteristics of porous materials. Shen et al.^23^ investigated the dependence of random open-cell foam models on relative density by using the Voronoi tessellation technique to generate 3D random porous models using porosity and cell shape irregularity as design parameters. Porosity, cell shape irregularity, strut cross-sectional area, and strut cross-sectional shape were used as design parameters by Li et al., to generate a 3D Voronoi porous model based on the Voronoi tessellation technique. They then examined the effect of the design parameters on the effective mechanical properties of the open-cell foam material^24^. Unlike the previous approach of generating 3D RVEs by assigning a cross-sectional area to each edge of a Voronoi polygon, Yang et al.^4^ propose to reduce the volume of a Voronoi polygon by using porosity as a design parameter and combine it with Boolean operations to generate 3D random porous RVEs. According to studies, the microstructure of porous materials has a significant effect on the mechanical properties of materials. By using specific parameter settings, the Voronoi diagram method can reflect the microscopic random structure of porous materials. However, the irregularity of pore shape is always correlated with the pore distribution of 3D RVE generated by traditional Voronoi diagram. By generating pore shapes and then utilizing a random technique to locate each pore's center points, this problem can be resolved. Li et al., generated a 2D square random porous model by using porosity and pore size irregularity as design parameters and a double-normal distribution algorithm to control the pore distribution and pore size, respectively. According to the findings, the effective Young’s modulus of porous materials rises as the average distance between pores increases, while the randomness of pore size has little bearing on the material’s Young’s modulus^25^. Generatingrandomly distributed closed-cell spherical pore RVEs with random dimensions using the RSA (Random Sequential Adsorption) algorithm, Tarantino et al. discovered that the model is isotropic^26^. The degree of anisotropy of the RVEs was discovered to be correlated with the pore aspect ratio by Anoukou et al.^27^, who improved the RSA algorithm to generate randomly distributed non-overlapping ellipsoidal pore RVEs with random shapes and sizes. There are numerous studies that are similar to this one^28–30^, but this type of modeling primarily generates pores with regular shapes (spherical, square, ellipsoidal, etc.) and regulates the random distribution of pore particles by regulating the minimum distance between them. It is difficult to impose constraints on a randomly distributed collection of points based on pore shape size in order to create an open-cell random porous model with low porosity. Therefore, this method is mostly used to generate RVEs with a closed-cell structure, but for porous reservoir self-lubricating media such as oiled bearings, the open porosity is particularly important to enhance the lubricating properties of the material^5, 31^, and a closed-cell structure is not suitable to describe the solid phase structure of oiled bearings^3^. Additionally, it was demonstrated that the irregularity of the cell shape has a negligible impact on the mechanical properties of porous materials at low porosity^4, 24^. The majority of studies have only looked at how porosity, pore morphology (shape, size, and orientation), and cell wall morphology (wall thickness and cross-sectional area) affect the mechanical properties of porous materials, but they have not looked into how random the pore distribution is.

To that end, this study proposes a new modeling method for porous solid-phase structures used in oil-bearing porous fluid storage self-lubricating media based on the Voronoi diagram method. Six design parameters, including porosity, number of pores, and pore morphology, are defined to generate fully open-cell, 3D porous materials with random distribution. The study divides pore morphology into pore size and pore distribution. To establish the relationship between the microstructure and macroscopic mechanical properties of porous materials and to discuss the effects of microstructure such as pore size and pore distribution on the mechanical properties of porous materials, the effective mechanical properties of RVEs with different design parameters were predicted using a computational homogenization method. Finally, specimens were produced using SLA 3D printing technology, and uniaxial compression tests were run to ensure the accuracy of the numerical calculations.

## RVE generation

The RVE is the smallest volume of a material at the microscopic level and must contain enough microstructural information while being sufficiently smaller in size than the macroscopic structure in order to accurately represent a material's properties at the macroscopic level. There are two common definitions of RVEs: (a) as a single cell in a periodic microstructure; and (b) as having enough microscopic components to satisfy statistical homogeneity and ergodicity^32^. The study makes the assumption that the material has a periodic structure. The steps involved in generating an RVE and the corresponding control parameters are as follows.

### The initial pore distribution

This study generates 2D Voronoi diagrams using the Voronoi irregularity $\alpha$ and the number of pores *N*. Then, the Voronoi diagrams that meet the requirements are screened using the number of pores *N*. Finally, the Voronoi polygons are scaled using the porosity $\rho$ to generate the initial pore distribution. The specific steps are as follows. First, the model is generated based on the Voronoi diagram. Voronoi diagrams are generated by setting a specified number of random points in the plane, i.e., nucleation points, then taking the vertical bisector of the line connecting two adjacent random points and trimming the resulting vertical bisector according to the principle of non-intersection of lines, thus dividing the plane into a series of convex polygons. In this case, the randomness of the distribution and shape of the Voronoi polygons is controlled by the minimum permissible distance ${\text{d}}_{0}$ between the nucleation points. Defining the distance between adjacent nucleation points of a perfectly regular two-dimensional Voronoi fovea as ${\text{d}}_{s}$. The definition of the irregularity of a 2D Voronoi diagram is shown in Eq. (1). At the same time, to reduce the number of mesh and their singularity, the minimum side length of the Voronoi polygon is controlled to be $0.375d_{0}$.

1 $$\alpha = 1 - \frac{{d_{0} }}{{d_{s} }},\;\;\left( {0 \le \alpha \le 1} \right)$$

**Figure 1:**
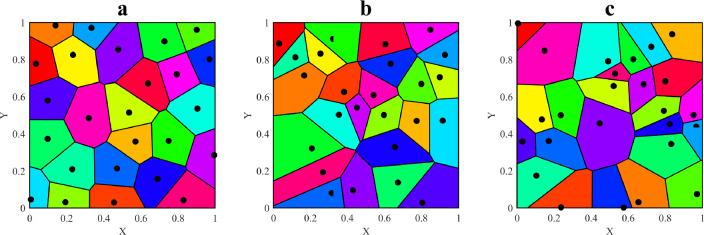
Voronoi diagram generated by different irregularities. (**a**) $\alpha = 0.2$. (**b**) $\alpha = 0.5$. (**c**) $\alpha = 0.8$.

As shown in Fig. 1, the smaller $\alpha$ means, the more regular the 2D Voronoi diagram.

Second, In order to reduce the restriction of the plane edge on the shape of the Voronoi polygon at the edge, the plane area set when generating the Voronoi diagram was expanded, and the number of nucleation points was increased proportionally. 2D Voronoi diagrams were continuously generated until the number of nucleation points within the desired range was equal to the required number of pores. Then take the Voronoi polygons whose nucleation points are within the expected range and use them to generate the RVE. For instance, if there are 25 nucleation points and the expected plane size is 1*1100 nucleation points are set up in the 2*2 plane to create the Voronoi graph, which is then used to extract the Voronoi polygons with nucleation points that fall within the middle of the 1*1 range.

**Figure 2:**
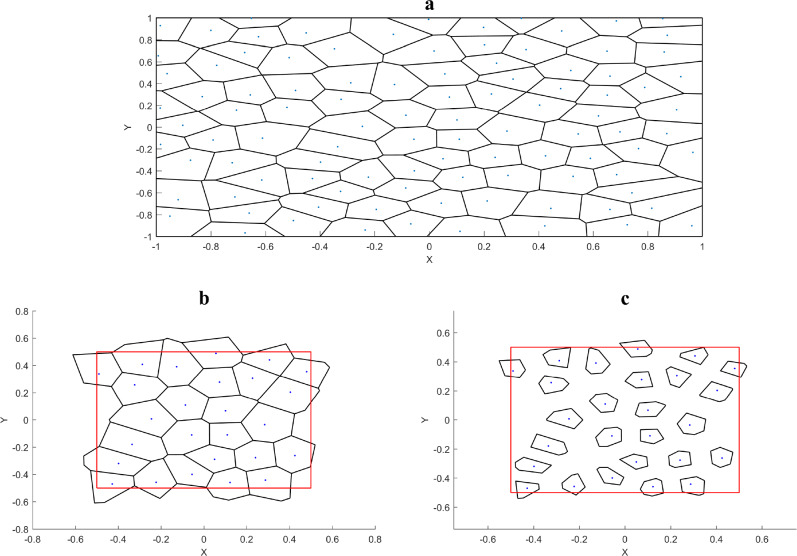
The process of generating the initial pore coordinates ($\rho = 0.25, \, N = 25, \, \alpha = 0.25$). (**a**) Voronoi diagram generated by scaling the planes according to the randomness of the pore distribution and the number of pores (where the red square indicates the desired plane range); (**b**) Voronoi polygon with the nucleation points within the desired plane range; (**c**) initial pore distribution obtained by scaling the Voronoi polygon according to the target porosity.

Finally, the Voronoi polygons are scaled with the nucleation points as the center according to the given porosity, as shown in Eq. (2), so that the pore volume can meet the porosity requirements. Figure 2 represents the process of obtaining the initial pore coordinates used to generate the RVE from the Voronoi diagram. Considering that the porosity of oiled bearings is generally 20–30%, the porosity of the RVEs generated in this paper is controlled to be around 25%.

2 $$\chi_{ij} = \sqrt \rho \left( {p_{ij} - p_{i0} } \right) + p_{i0}$$

where $\sqrt \rho$ is the porosity design parameter and $\rho$ denotes the material porosity; $i = x,y$ denotes the reference axis of the Cartesian coordinate system; $j = 1,2, \ldots ,n$, *n* denotes the number of vertices of the Voronoi polygon; $\chi_{ij}$ represents the coordinates of the vertices of the Voronoi polygon that is ultimately used to generate the RVE.;$p_{i0}$, $p_{ij}$ denotes the coordinates of the nucleation points and vertices of the Voronoi polygon, respectively, while specifying $p_{z0} = \chi_{zj} = 0$.

### Design parameters in the *x*, *y* direction

**Figure 3:**
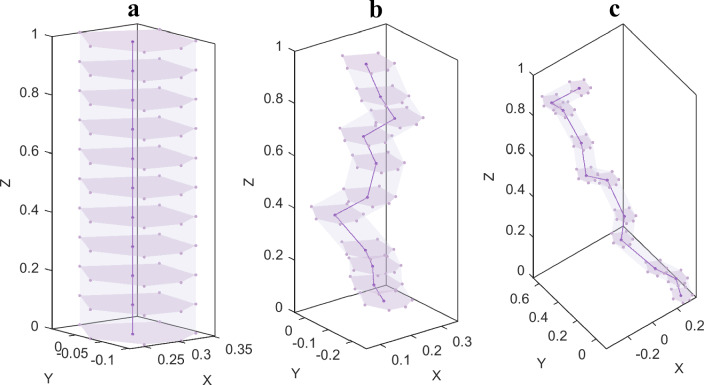
Examples of pipes that have been produced using various *x, y* design parameters. The dark purple line depicts the growth curve of the pore nucleation points when the pipe is generated using the specified* x* and *y* design parameters; the dark purple points are the nucleation points, the lavender points are the vertices of each pore particle determined by the aforementioned equation, the lavender surface is the pore cross-section enclosed by the resulting vertices, and the nearly transparent lavender surface is the pore wall. (**a**) $\varpi_{{\text{x}}} = 0,\varpi_{y} = 0$; (**b**) $\varpi_{x} = 1,\varpi_{y} = 1$; (**c**) $\varpi_{x} = 2,\varpi_{y} = 2$.

The pores of porous materials used to make porous fluid storage self-lubricating bearings can be visualized as a collection of overlapping, irregularly shaped curving pipelines that are each composed of a number of stacked pore particles. In this investigation, the pores were divided into various pipes. Assuming that each pipe is made up of a stack of ten irregular prismatic pore particles and that the porous material grows linearly along the positive *z-*axis depending on the initial pore shape, let the governing equation for its growth trajectory be as stated in Eq. (3).

3 $$p_{i0}^{\ell + 1} = p_{i0}^{\ell } + \omega_{i}^{\ell } l_{z}^{e} ,\;i = x,y$$

4 $$p_{i0}^{\ell + 1} = \left( {\ell + 1} \right)l_{z}^{e} ,\;i = z$$

5 $$\chi_{ij}^{\ell + 1} = \chi_{ij}^{\ell } + \omega_{i}^{\ell } l_{z}^{e} ,\;i = x,y$$

6 $$\chi_{ij}^{\ell + 1} = \left( {\ell + 1} \right)l_{z}^{e} ,\;i = z$$

7 $$l_{z}^{e} = \frac{{l_{z} }}{10}$$

8 $$\omega_{i}^{\ell } = \varpi_{i} a_{i}^{\ell } ,\;i = x,y$$

where $\ell = 0,1, \ldots ,9$,and $\chi^{\ell + 1} , \, p^{\ell + 1}$ denotes the coordinates of the vertex and nucleation of the ($\ell + 1$)th pore particle(from the bottom to the top of the RVE); $l_{z} ,l_{z}^{e}$ denotes the size of the RVE in the* z* direction and the height of the pore particles respectively; $\varpi_{i}$ denotes the design parameters in the* x* and *y* directions, the larger $\varpi_{i}$, the greater the randomness of the spatial distribution of the pore; $a_{i}$ is a set of random numbers uniformly distributed on $\left( { - \;1,1} \right)$; while the corresponding coordinates of the pipe for $\ell = 0$ are:$p_{i0}^{0} = p_{i0} , \, \chi_{ij}^{0} = \chi_{ij}$. The entire pore pipe model can be generated by connecting the corresponding points once each pore particle’s top surface coordinates have been generated, as illustrated in Fig. 3.

### Design parameters in the* z* direction

Considering that each section of each pore in a random porous material has a different radius, the vertex function of each pore particle can be determined by taking the nucleation point of each pore particle as the pole, setting up a local polar coordinate system, selecting the pole diameter $r_{j0}^{\ell }$ corresponding to $\chi_{ij}^{\ell }$ as the initial pore diameter, and varying the initial pore diameter within a specific range in accordance with the *z* directional design parameters.

9 $$r_{j}^{\ell } = r_{j0}^{\ell } + \omega_{r}^{\ell } r_{j0}^{\ell } ,\;\;\,\ell = 0,1, \ldots ,10$$

10 $$\omega_{rj}^{\ell } = \varpi_{r} a_{r}^{\ell }$$

**Figure 4:**
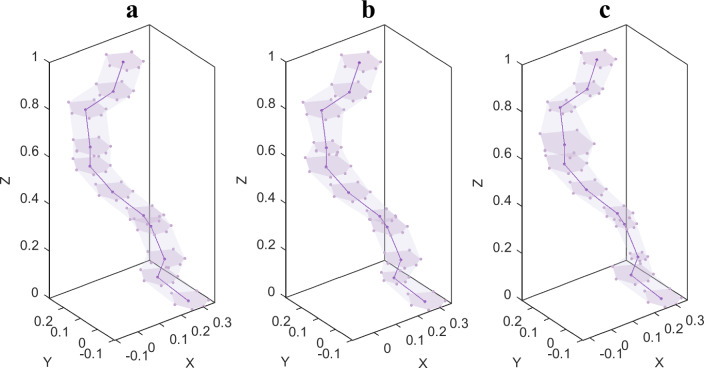
(**a**–**c**) Examples of pipes that have been produced using various x, y design parameters ($\varpi_{x} = \varpi_{y} = 1$). The dark purple line depicts the growth curve of the pore nucleation points when the pipe is generated using the specified* x* and *y* design parameters; the dark purple points are the nucleation points, the lavender points are the vertices of each pore particle determined by the aforementioned equation, the lavender surface is the pore cross-section enclosed by the resulting vertices, and the nearly transparent lavender surface is the pore wall. (**a**) $\varpi_{r} = 0$; (**b**) $\varpi_{r} = 0.3$; (**c**) $\varpi_{r} = 0.6$.

$\varpi_{r}$ is the *z* directional design parameter, with a larger $\varpi_{r}$ indicating greater randomness in the pore size of the pore pipe; $a_{r}$ is a set of random numbers that are evenly distributed on $\left( { - \;1,1} \right)$. As seen in Fig. 4, this reconstructs the pore wall to generate a pore pipe model that accounts for the* z* direction design parameters.

### Periodicity of RVE

As previously mentioned, the directional design of the pore can be accomplished and, using Boolean operations, the target RVE can then be obtained given the RVE dimensions and the design parameters $N,\alpha ,\rho ,\varpi_{x} ,\varpi_{y} ,\varpi_{r}$. However, because the porous material in this study is assumed to be periodic, the RVE boundary must adhere to the same standards for continuity and relative surface structure. The portion of a pore outside the RVE that passes through the RVE's boundary must be transferred to the relative boundary of the RVE without changing orientation. As a result, the RVE's period types are split into two categories, which are detailed below.

#### Periodicity in the *x, y* direction

**Figure 5:**
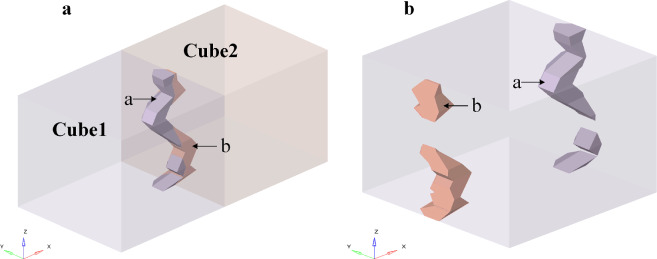
Pores pass through the boundary surface in the* x* direction. (**a**) Two RVEs share a pore, with Cube1 denoting the initial RVE and Cube2 denoting the adjacent RVE; a is the portion of the pore that lies within the initial RVE; b denotes the portion of the pore that lies within the adjacent RVE; (**b**) the pore beyond the boundary is transferred to the corresponding relative surface of the initial RVE.

**Figure 6:**
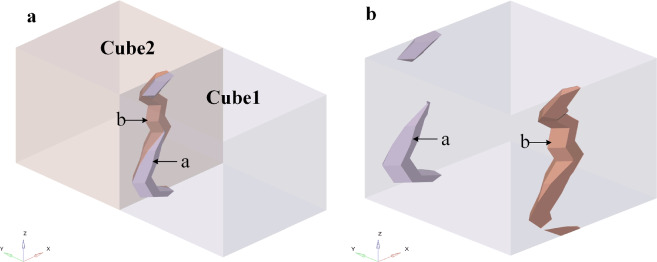
Pores pass through the boundary surface in the *y* direction. (**a**) Two RVEs share a pore, with Cube1 denoting the initial RVE and Cube2 denoting the adjacent RVE; a is the portion of the pore that lies within the initial RVE; b denotes the portion of the pore that lies within the adjacent RVE. (**b**) The pore beyond the boundary is transferred to the corresponding relative surface of the initial RVE.

The pore may go through four faces as it crosses the RVE boundary (positive *x*, negative *x*, positive *y*, and negative* y* faces). As shown in Fig. 5a, the portion of the pore beyond the RVE will enter the neighboring RVE by the negative *x* face as it passes through the positive *x* face of the RVE. As a result, the portion of the pore that is outside the original RVE is cut off and moved to the original RVE's negative *x* plane (as illustrated in Fig. 5b). The portion of the pores that extends past the RVE is transferred to the negative *y* plane as they pass through the positive *y* plane, as seen in Fig. 6.

**Figure 7:**
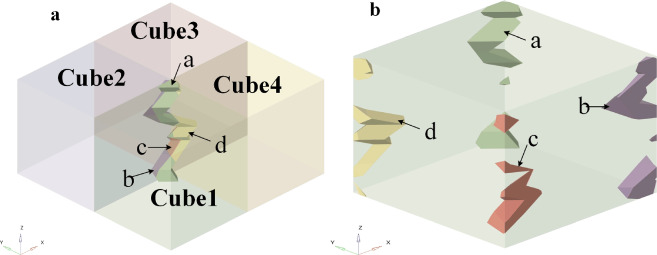
The pore passes over one edge and two faces. (**a**) Four RVEs share a pore. Cube1 denotes the initial RVE; Cube2, Cube3, and Cube4 denote adjacent RVEs; a is the portion of the pore located within the initial RVE; b, c, and d denote portions of the pore located within adjacent RVEs, respectively; (**b**) the pore beyond the boundary is transferred to the corresponding relative surface of the initial RVE.

Similarly, as the pores cross the RVE’s edge, the pore simultaneously enters three adjacent RVEs. Trimming and transfer to the corresponding opposite face are done with the portion that extends past the RVE. For instance, when the pore traverses the negative *x–y* edge, the portion of the pore that traverses the positive* x*, positive *y*, and positive the *x–y* edge is clipped and moved to the respective negative *x*, negative *y*, and negative *x–y* edges, as shown in Fig. 7.

#### Periodicity in the *z* direction

**Figure 8:**
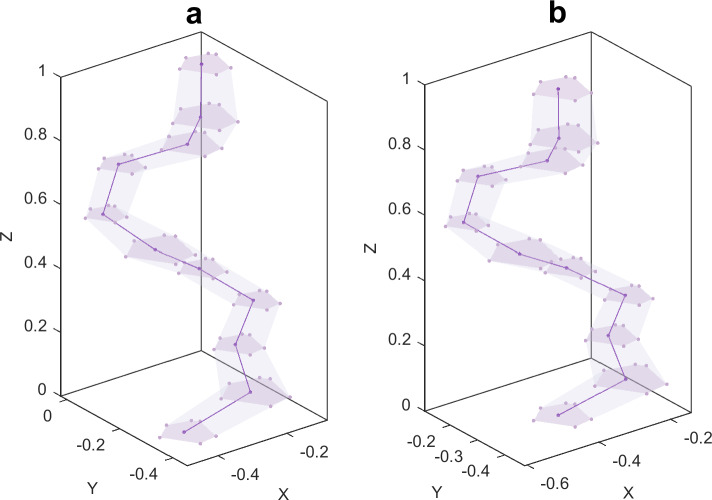
Constrained pores ($\varpi_{x} = \varpi_{y} = 1.5$,$\varpi_{r} = 0.3$) are applied. (**a**) The pore before restraint is applied. (**b**) The pore after the application of the constraint.

The positive* z* plane and the negative *z* plane of the RVE must both have the same pore distribution in order for the RVE to achieve periodicity in both the positive *z* plane and the negative *z* plane. As a result, the following constraint is placed on $a_{x} ,a_{y} ,{\text{ and }}a_{r}$ in this study. Figure 8 illustrates the pores once the constraint is in place.

11 $$\sum\limits_{\ell = 1}^{10} {a_{i}^{\ell } } = 0,\;\;i = x,y$$

12 $$a_{r}^{10} = 0$$

### Corrected porosity

Although the single pore model is obtained by volume scaling based on a given porosity, the staggering and overlapping of pores, the randomness of pore size, and the realization of periodic structures will all have an impact on the volume of the overall pores generated. Therefore, after generating the overall pores, the pore model is reconstructed according to Eq. (13) to achieve the purpose of correcting the porosity of the RVE.

13 $$\hat{r}_{j}^{\ell } = \sqrt {\rho V_{RVE} /V_{r} } r_{j}^{\ell } , \, \ell = 0,1, \ldots 10.$$

where $\hat{r}_{j}^{\ell }$ represents the polar radius corresponding to the pore vertex after correction.

## Calculation implementation

This study uses a computational homogenization method to perform a finite element analysis of the mechanical properties of the porous material RVE.

### Computational homogenization method

At sufficiently tiny sizes, any material can be thought of as being non-homogeneous, although at macroscopic dimensions, one often considers the statistical homogeneity of the material. The homogenization method uses the fine-scale strain field of a material to solve for the macroscopic effective properties of that material. The homogenization calculations below are based on biphasic composites since porous materials can be thought of as composites of a base material plus air.

According to homogenization theory^33^, the average stress $\overline{\sigma }_{ij}$ and the average strain $\overline{\varepsilon }_{ij}$ are defined as:

14 $$\overline{\sigma }_{ij} = \frac{1}{V}\int_{V} {\sigma_{ij} \left( x \right)dV} ,\;\;\overline{\varepsilon }_{ij} = \frac{1}{V}\int_{V} {\varepsilon_{ij} \left( x \right)dV}$$

where *V* is the volume of the RVE, $\sigma_{ij} \left( x \right)$ and $\sigma_{ij} \left( x \right)$ is the stress and strain states at any point, respectively. The effective stiffness $\overline{C}_{ijkl}$ and the effective flexibility $\overline{S}_{ijkl}$ is defined as:

15 $$\overline{\sigma }_{ij} = \overline{C}_{ijkl} \overline{\varepsilon }_{kl}$$

16 $$\overline{S}_{ijkl} = \overline{C}_{ijkl}^{ - 1}$$

In order to calculate the average stress and average strain in a multiphase material, Hill^34^ introduced a phase average concentration for the various phases in the material. The particular calculation is displayed below.

17 $$\overline{\sigma }_{ij} = \frac{1}{V}\left( {\int_{{V_{f} }} {\sigma_{ij}^{f} dV_{f} + \int_{{V_{m} }} {\overline{\sigma }_{ij}^{m} dV_{m} } } } \right)$$

18 $$\overline{\sigma }_{ij}^{f} = \frac{1}{{V_{f} }}\int_{{V_{f} }} {\sigma_{ij}^{f} dV_{f} } ,\;\;\overline{\sigma }_{ij}^{m} = \frac{1}{{V_{m} }}\int_{{V_{m} }} {\sigma_{ij}^{m} dV_{m} }$$

19 $$\overline{\sigma }_{ij} = v_{f} \overline{\sigma }_{ij}^{f} + v_{m} \overline{\sigma }_{ij}^{m}$$

where $f$,$m$ denotes air and matrix material respectively, and $v_{f} ,v_{m}$ denotes the volume fraction of air and matrix material, respectively. Similarly, the average strain can be expressed as:

20 $$\overline{\varepsilon }_{ij} = v_{f} \overline{\varepsilon }_{ij}^{f} + v_{m} \overline{\varepsilon }_{ij}^{m}$$

Equation (18) can be stated as follows based on the constitutive relationship of the composite phases:

21a $$\overline{\sigma }_{ij}^{f} = \frac{1}{{V_{f} }}\int_{{V_{f} }} {C_{ijkl}^{f} \left( x \right)} \varepsilon_{kl}^{f} \left( x \right)dV_{f}$$

21b $$\overline{\sigma }_{ij}^{m} = \frac{1}{{V_{m} }}\int_{{V_{m} }} {C_{ijkl}^{m} \left( x \right)\varepsilon_{kl}^{m} \left( x \right)dV_{m} }$$

where each phase's stiffness matrix $C_{ijkl}$ is regarded as a constant term. Therefore:

22a $$\overline{\sigma }_{ij}^{f} = C_{ijkl}^{f} \frac{1}{{V_{f} }}\int_{{V_{f} }} {\varepsilon_{kl}^{f} \left( x \right)dV_{f} } = C_{ijkl}^{f} \overline{\varepsilon }_{ij}^{f}$$

22b $$\overline{\sigma }_{ij}^{m} = C_{ijkl}^{m} \frac{1}{{V_{m} }}\int_{{V_{m} }} {\varepsilon_{kl}^{m} \left( x \right)dV_{m} } = C_{ijkl}^{m} \overline{\varepsilon }_{ij}^{m}$$

The displacement boundary condition is applied in this investigation. Thus, the average strain of the RVE can be expressed using the divergence theorem, as shown in Eq. (23). And Eq. (24) will establish a relationship between the local strain and the average strain for any point in the RVE.

23 $$\overline{\varepsilon }_{ij} = \frac{1}{V}\int_{\Gamma } {\frac{1}{2}\left( {\overline{u}_{i} \overline{\eta }_{j} + \overline{u}_{j} \overline{\eta }_{i} } \right)} {\text{d}}\Gamma$$

24 $$\varepsilon_{ij}^{f} \left( x \right) = A_{ijkl}^{f} \left( x \right)\overline{\varepsilon }_{kl} ,\;\;\varepsilon_{ij}^{m} \left( x \right) = A_{ijkl}^{m} \left( x \right)\overline{\varepsilon }_{kl}$$

where, $\Gamma$ represents the surface of the RVE, $\overline{\eta }$ is the normal direction of the RVE boundary, $\overline{u}$ is the displacement, and $A_{ijkl}$ is the tensor of the strain concentration factor. Equation (25) is obtained by integrating Eq. (24) and taking the volume average.

25 $$\overline{\varepsilon }_{ij}^{f} = \left[ {\int_{{V_{f} }} {A_{ijkl}^{f} \left( x \right){\text{d}}V_{f} } } \right]\overline{\varepsilon }_{kl} = \overline{A}_{ijkl}^{f} \overline{\varepsilon }_{kl} , \, \overline{\varepsilon }_{ij}^{m} = \left[ {\int_{{V_{f} }} {A_{ijkl}^{m} \left( x \right){\text{d}}V_{f} } } \right]\overline{\varepsilon }_{kl} = \overline{A}_{ijkl}^{m} \overline{\varepsilon }_{kl}$$

where $\overline{A}$ is the phase's average strain concentration factor. Equations (22) and (25) can be substituted for Eq. (19) to determine the global average stress as follows:

26 $$\overline{\sigma }_{ij} = \left[ {v_{f} C_{ijkl}^{f} \overline{A}_{klmn}^{f} + v_{m} C_{ijkl}^{m} \overline{A}_{klmn}^{m} } \right]\overline{\varepsilon }_{mn}$$

Equation (26), when compared to Eq. (15), yields the following as the composite's effective stiffness matrix:

27 $$\overline{C}_{ijkl} = v_{f} C_{ijkl}^{f} \overline{A}_{klmn}^{f} + v_{m} C_{ijkl}^{m} \overline{A}_{klmn}^{m}$$

### Computational implementation of RVE

**Figure 9:**
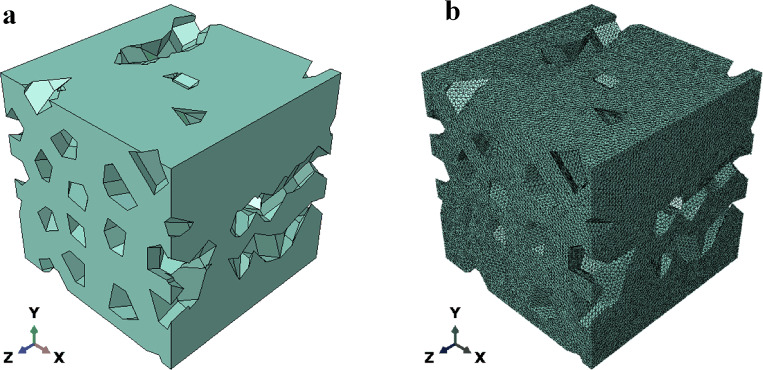
The RVE design parameters are $N = 16, \, \alpha = 0.25, \, \rho = 0.25, \, \varpi_{{\text{x}}} = 1, \, \varpi_{y} = 1, \, \varpi_{r} = 0$. (**a**) The RVE geometry model generated in Abaqus; (**b**) the RVE meshed.

The pores and a square of a specific size were generated in Abaqus using a Python script in accordance with the method for generating pores described in sections “The initial pore distribution”–“Design parameters in the z direction”. The pores penetrate the square. Subsequently, the corresponding pores were treated periodically in accordance with the method in section “Periodicity of RVE”. The pore Instances were then combined. Finally, a Boolean operation was used to obtain the target RVE, as shown in Fig. 9a.

### Finite element analysis of the RVE

All of the finite element analyses in this study were carried out by combining Python and Abaqus. Assume that the base material of the solid is homogeneous, isotropic and linearly elastic. Using the normalization method, take the RVE size to be $1 \times 1 \times 1{,}$ Young's modulus $E_{{\text{s}}} = 1$ ($E_{s}$ is linearly proportional to the effective stiffness) and Poisson’s ratio $\nu_{s} = 0.34.$ The finite element analysis was performed using a ten-node tetrahedral element (C3D10), as shown in Fig. 9b.

The superposition principle was used to impose displacement boundary conditions as a combination of six pure strain components, and finite element calculations were carried out for each boundary condition. Then, from the ODB file, the strain components $\left\{ {\varepsilon_{11} ,\varepsilon_{22} ,\varepsilon_{33} ,\varepsilon_{23} ,\varepsilon_{13} ,\varepsilon_{12} } \right\}^{T}$ at each element's integration point, the element volume, and the local orientation of each element were extracted. Finally, the effective performance of the RVE was calculated using this data (as explained in “Computational homogenization method”).

## Results and discussion

Because a large portion of previous research^4–6, 35^, concentrated on how porosity affected the mechanical properties of porous materials, the findings almost universally indicated that mechanical properties of porous materials decreased as porosity $\rho$ increased. As a result, this study will use a controlled variable approach to discuss the effects of the number of pores *N*, the initial pore distribution uniformity $\alpha$, the pore growth randomness $\varpi_{{\text{x}}} ,\varpi {}_{y}$ and the pore size randomness $\varpi_{r}$ on the effective mechanical properties of porous materials.

### Mesh convergence study

**Figure 10:**
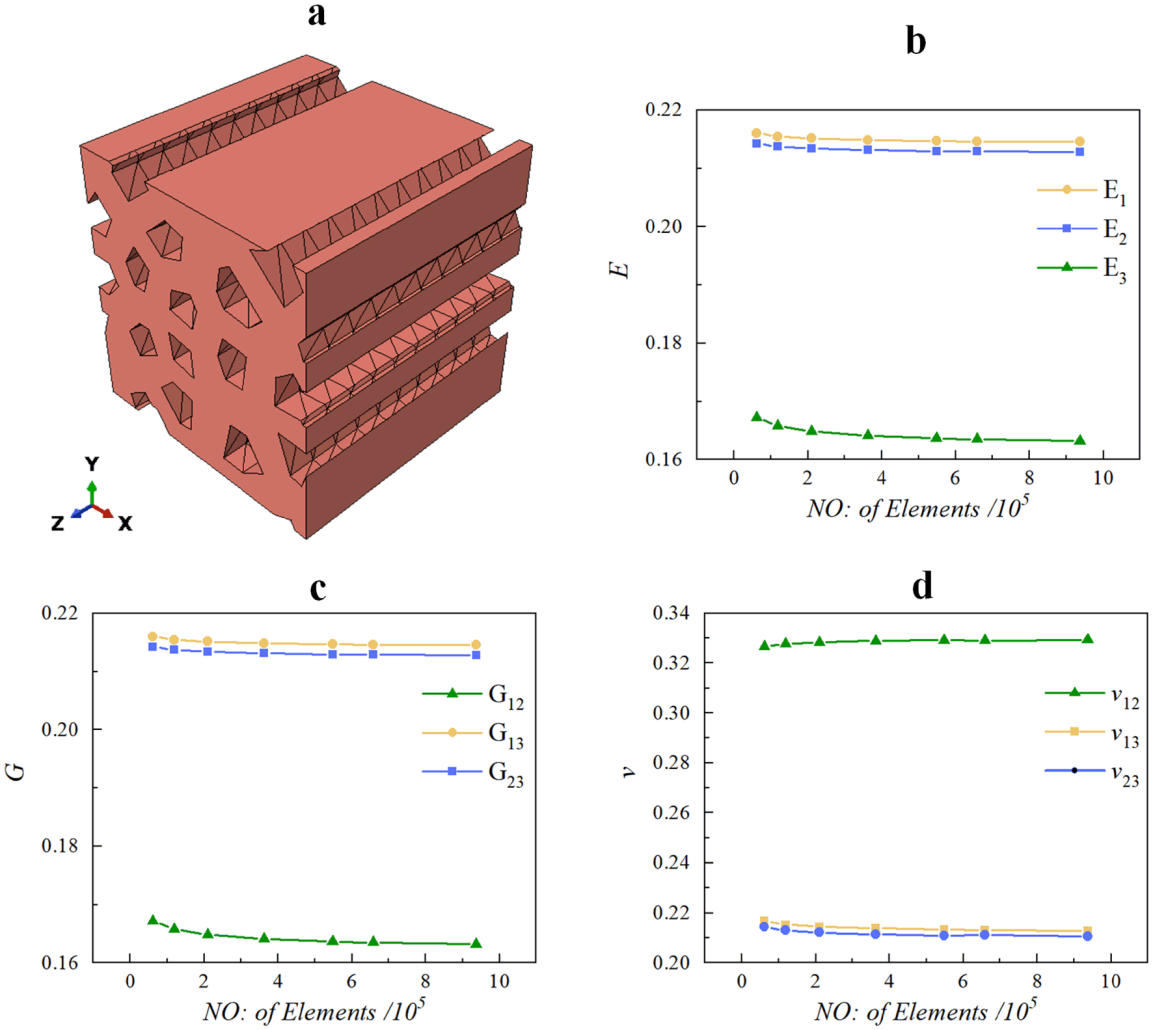
(**a**) An RVE geometry model with the following design parameters $\rho = 0.25, \, \alpha = 0.25{, }\varpi_{x} = \varpi_{y} = \varpi_{r} = 0, \, N = 16$; (**b**–**d**) convergence of effective performance with the number of elements in an RVE.

By examining the numerical results for the same RVE with various element numbers, the mesh convergence study was carried out. For the RVE, shown in Fig. 10a, with design parameters $\rho = 0.25, \, \alpha = 0.25, \, \varpi_{x} = \varpi_{y} = \varpi_{r} = 0, \, N = 16$. The number of elements are varied from $6 \times 10^{4}$ to $1 \times 10^{6}$. The results are displayed in Fig. 10b–d. When there are more than $5 \times 10^{5}$ elements, it is clear that the numerical results converge. As a result, to discretize the remaining RVE models, mesh sizes with element counts of $5 \times 10^{5}$ and higher are used.

In addition, the directions 1, 2 and 3 are used to indicate directions *x, y* and* z*, respectively. The stress and strain vectors in the resulting constitutive model are $\left\{ {\sigma_{11} ,\sigma_{22} ,\sigma_{33} ,\sigma_{23} ,\sigma_{13} ,\sigma_{12} } \right\}^{T}$ and $\left\{ {\varepsilon_{11} ,\varepsilon_{22} ,\varepsilon_{33} ,\varepsilon_{23} ,\varepsilon_{13} ,\varepsilon_{12} } \right\}^{T}$, respectively, so the components of the effective stiffness tensor are derived by mapping these tensors one to one. The following engineering constants are $E_{1}$, $E_{2}$, $E_{3}$, $G_{12}$, $G_{13}$, $G_{23}$, $v_{12}$ and $v_{23}$.

### Effects of the number of pores

The effect of the number of pores *N* on the mechanical properties of the RVE was analyzed for a columnar pore RVE (i.e., $\varpi_{{\text{x}}} = 0, \, \varpi_{y} = 0, \, \varpi_{r} = 0$) with porosity design parameter $\rho = 0.25$ and Voronoi diagram irregularity $\alpha = 0.25$. Five independent sets of nucleation points $p_{i0}$ ($i = x, \, y$) were used to generate five porous material models for each value of *N*, i.e.,* N* = 4, 9, 16 and 25. In addition, the porosity of the RVE generated in the study is all within the range of $\left( {25 \pm 0.5} \right)\%$.

**Figure 11:**
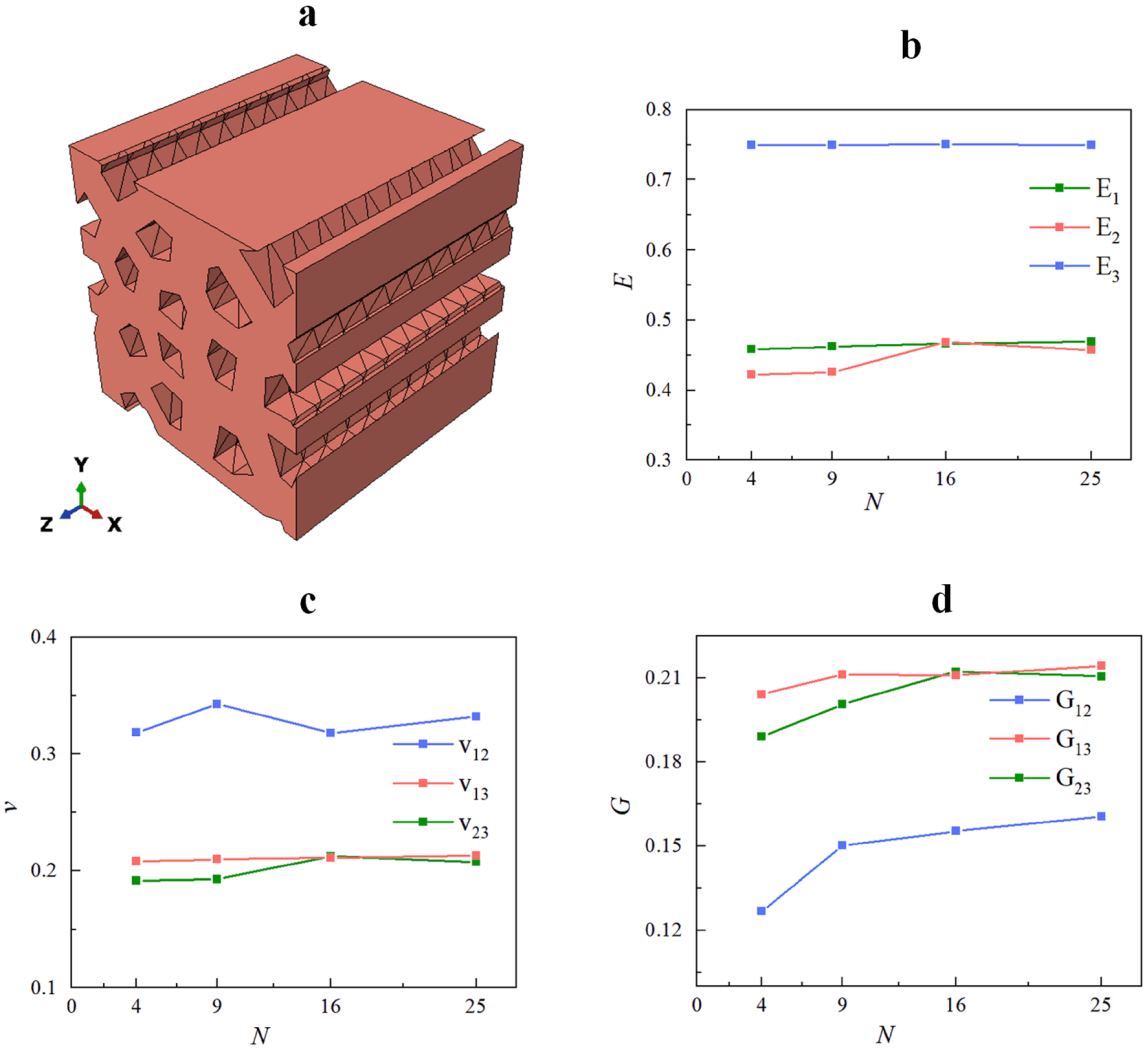
(**a**) An RVE geometry model with design parameters $\rho = 0.25, \, N = 16, \, \alpha = 0.25, \, \varpi_{x} = \varpi_{y} = \varpi_{r} = 0$. (**b**–**d**) Effects of pore number on RVE effective mechanical properties.

Figure 11 shows one of the RVEs generated in this section and the average value of the effective engineering constants at different *N* values. The corresponding relative standard deviation (RSD) is shown in Table 1. Figure 11 indicates that for a prismatic RVE, its Young’s modulus in the *x* and *y* direction $E_{1} , \, E_{2}$ are close (maximum relative difference of 8%), the Poisson’s ratio in the *XZ* and *YZ* planes $v_{13} , \, v_{23}$ are close (maximum relative difference of 8%) and shear modulus in the *XZ* and *YZ* planes $G_{13} , \, G_{23}$ are close (maximum relative difference of 8%). The effect of the number of pores *N* on the Young’s modulus $E_{3}$ in the *z* direction of the RVE can be ignored.

**Table 1:** Relative standard deviation in effective properties for porous materials with different *N* (unit :%).

*N*	$E_{1}$	$E_{2}$	$E_{3}$	$G_{12}$	$G_{13}$	$G_{23}$	$v_{12}$	$v_{13}$	$v_{23}$
4	7.49	21.04	0.12	3.89	2.79	13.15	28.92	7.55	21.02
9	2.05	13.09	0.03	12.27	2.02	6.29	8.94	2.05	13.08
16	3.43	3.73	0.23	5.13	2.83	1.88	5.33	3.27	3.89
25	3.22	1.42	0.11	4.26	1.63	1.46	3.06	3.14	1.47

From Table 1, it can be seen that when *N* = 16 and *N* = 25, the relative standard deviation of the effective engineering constants of the RVE is less than 15%. This indicates that the effective engineering constants of the RVE are stable when *N* = 9, *N* = 16 and *N* = 25. However, when* N* = 4, the effective modulus of the RVE fluctuates greatly, with the maximum relative standard deviation of its effective engineering constants being 29%. Babu discovered that the mechanical properties of a material are influenced by the projected area of the fibers in the corresponding direction when he looked into the effective mechanical properties of fiber composites^36^. When there are few pores, the position of the pores affects the projected area of the pores on the *XZ* and *YZ* planes. The relative projected area of the pores on the *XZ* and *YZ* planes approaches 100% as the number of pores rises. As a result of the randomness of the pore locations, when the number of pores is low, the effective engineering constants $E_{1} , \, E_{2} , \, G_{12} , \, G_{13} , \, G_{23} , \, v_{12} , \, v_{13} ,{\text{ and }}v_{23}$ fluctuate greatly.

### Effect of inhomogeneity of initial pore distribution

**Figure 12:**
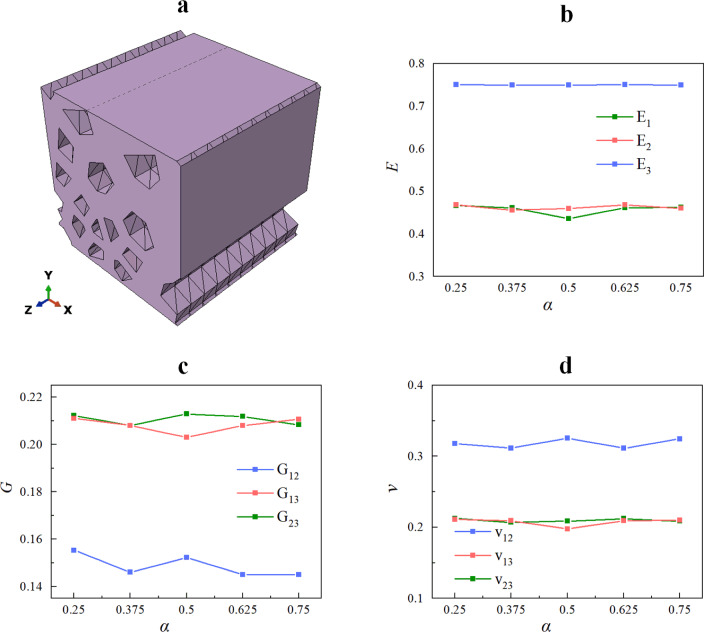
(**a**) An RVE geometry model with design parameters $\rho = 0.25, \, N = 16, \, \alpha = 0.5, \, \varpi_{x} = \varpi_{y} = \varpi_{r} = 0$. (**b**–**d**) Effects of pore irregularity on RVE effective mechanical properties.

Based on the research in section “Effects of the number of pores”, in order to reduce the effect of the number of pores on the results, the effect of the initial pore distribution inhomogeneity $\alpha$ on the mechanical properties of the RVE was analyzed for a columnar pore RVE (i.e.$\varpi_{x} = 0, \, \varpi_{y} = 0, \, \varpi_{r} = 0$) with $N = 16$. For each value of $\alpha$, five independent sets of nucleation points $p_{i0}$ ($i = x,y$) were used to generate five porous material models, respectively. Figure 12 shows one of the RVEs generated in this section and the average value of the effective engineering constants of the RVE at different $\alpha$ values. Table 2 shows the relative standard deviation of the corresponding effective properties.

**Table 2:** Relative standard deviation in effective properties for porous materials with different $\alpha$ (unit: %).

$\alpha$	$E_{1}$	$E_{2}$	$E_{3}$	$G_{12}$	$G_{13}$	$G_{23}$	$v_{12}$	$v_{13}$	$v_{23}$
0.25	3.43	3.73	0.23	5.13	2.83	1.88	5.33	3.27	3.89
0.375	7.20	2.95	0.16	6.82	5.18	2.28	7.08	7.08	3.04
0.5	5.55	4.67	0.07	4.39	2.57	1.79	5.81	5.60	4.65
0.625	6.68	3.75	0.04	10.44	4.01	3.18	7.86	6.71	3.75
0.75	6.34	9.32	0.03	3.85	2.68	3.94	5.62	6.33	9.31

Figre 12 shows that $\alpha$ has little effect on the effective mechanical properties of the RVE. At the same time, similar conclusions can be drawn for the mechanical properties of a columnar RVE as in section “Effects of the number of pores”. Observing Table 2, it can be seen that the relative standard deviation of the effective properties predicted at different $\alpha$ values is all less than 15%. This indicates that the numerical discretization is small and the numerical results are stable. In addition, it can be seen from Figs. 11 and 12 that the Young’s modulus in the *z* direction of a columnar RVE is independent of the distribution of pores in the *XY* plane and remains around 0.75.

Li discovered that the inhomogeneity of the strut cross-sectional area decreased the mechanical properties of foams^24^. At the same time, changes in the relative projection area of the pores and the implementation of periodic structures will also have an impact on the mechanical properties of the RVE. These factors caused fluctuations in the mechanical properties of RVE, but Table 3 shows that this fluctuation is acceptable.

**Table 3:** Relative standard deviation in effective properties for porous materials with different $\varpi_{r}$(unit : %).

$\varpi_{r}$	$E_{1}$	$E_{2}$	$E_{3}$	$G_{12}$	$G_{13}$	$G_{23}$	$v_{12}$	$v_{13}$	$v_{23}$
0	3.43	3.73	0.23	5.13	2.83	1.88	5.33	3.27	3.89
0.2	8.37	5.90	0.08	6.84	3.34	2.46	8.26	8.39	5.91
0.4	2.22	6.76	0.20	4.45	2.24	2.46	10.55	2.27	6.86
0.6	2.09	3.37	0.84	4.13	0.94	1.55	4.83	1.81	3.60
0.8	3.75	2.95	1.02	6.66	4.04	1.72	8.21	4.10	2.80
1	3.16	5.40	1.45	5.78	3.08	2.90	3.29	4.46	5.16

### Effect of randomness in pore size

**Figure 13:**
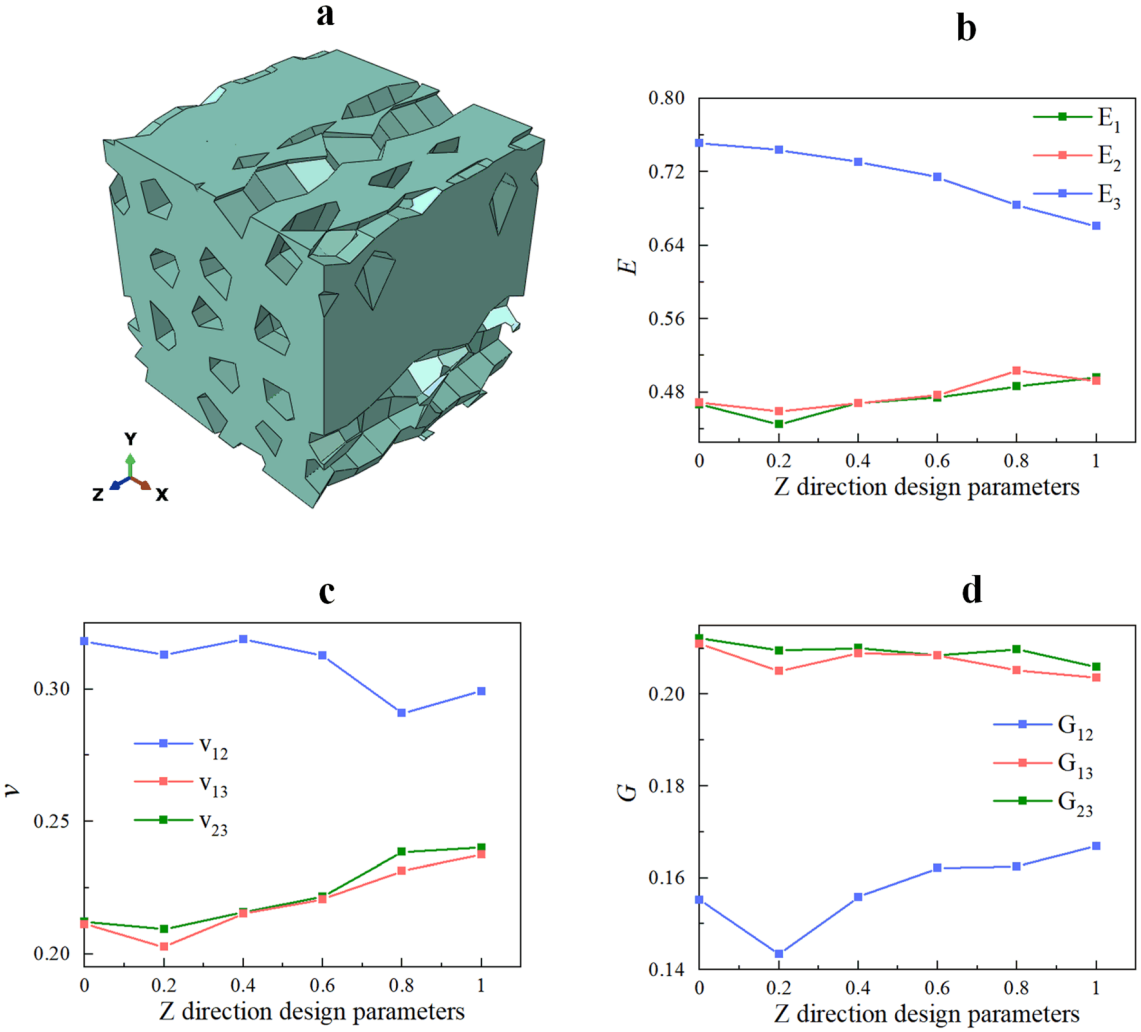
(**a**) An RVE geometry model with design parameters $\rho = 0.25, \, N = 16, \, \alpha = 0.25, \, \varpi_{x} = \varpi_{y} = 0 , \, \varpi_{r} = 0.8$; (**b**–**d**) effects of randomness in pore size on RVE effective mechanical properties.

To reduce the effect of other factors on the mechanical properties of the RVE, the effect of pore size randomness ($\varpi_{r}$) was studied for a porous material model with $\alpha = 0.25$, $N = 16$, $\rho = 0.25$, $\varpi_{x} = 0$, $\varpi_{y} = 0$. Five porous material models were generated using five independent sets of nucleation points $p_{i0}$ $\left( {i = x,y} \right)$ and random values $a_{r}^{\ell k} (\ell = 0,1,...,9;k = 1,2,...,N)$ for each $\varpi_{r}$ value, i.e.,$\varpi_{r} = 0.2,0.4,0.6, \, 0.8, \, 1$. Figure 13 shows one of the RVEs generated in this section and the average value of the effective engineering constants at different $\varpi_{r}$ values. The corresponding relative standard deviation (RSD) is shown in Table 3.

**Figure 14:**
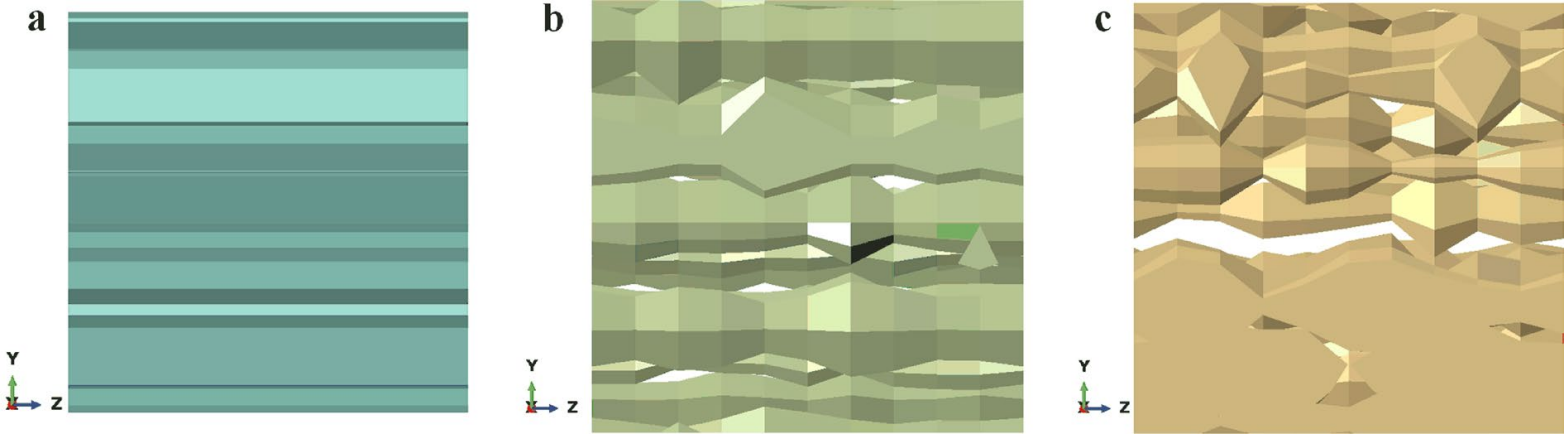
The projection of the pore on the *YZ* plane with different $\varpi_{r}$. (**a**) $\varpi_{r} = 0$; (**b**) $\varpi_{r} = 0.4$; (**c**) $\varpi_{r} = 0.8$.

Figure 13 shows that, on average, the effect of $\varpi_{r}$ on the mechanical properties of the RVE is small. As $\varpi_{r}$ increases further, $E_{3}$ and $v_{12}$ decrease, $E_{1}$,$E_{2}$,$G_{12}$,$v_{13}$ and $v_{23}$ increase. The effect of $\varpi_{r}$ on $G_{13}$ and $G_{23}$ is negligible. This occurs because, as $\varpi_{r}$ increases, the pore size inhomogeneity of the porous material increases and the projected area of the pores decreases on the *XZ* and *YZ* planes (as shown in Fig. 14) and increases on the *XY*. Therefore, the randomness of the pore size has an enhancing effect on the mechanical properties of the RVE in the* x* and *y* directions and a weakening effect on the mechanical properties of the pores in the *z* direction, and this effect increases with the randomness of the pore size increases. Equation (9) shows that $\varpi_{r}$ leads to proportional scaling of the pore cross-section with respect to the *z* direction, resulting in a close change in the projected area of the pore on the *XZ* and *YZ* planes, leading to a close effect of $\varpi_{r}$ on the mechanical properties of the porous material in the *x* direction and *y* direction In addition, the effect of $\varpi_{r}$ on the effective engineering constants of the RVE is not significant. When $\varpi_{r} = 1$, the average values of $E_{1}$,$E_{2}$,$G_{12}$,$v_{13}$ and $v_{23}$ are 6%, 5%, 8%, 12%, and 13% larger than when $\varpi_{r} = 0$, respectively. $E_{3}$ and $v_{12}$ are relatively reduced by 12% and 6%, respectively. In terms of Young’s modulus, as $\varpi_{r}$ increases, the increase in $E_{1}$ and $E_{2}$ is smaller than the decrease in $E_{3}$. From Table 3, it can be observed that the relative standard deviation of the effective stiffness of the RVEs generated at different $\varpi_{r}$ values is less than 15%, indicating that the method of generating RVEs and predicting effective stiffness in this study is repeatable.

## .

### Effect of pore growth randomness

**Figure 15:**
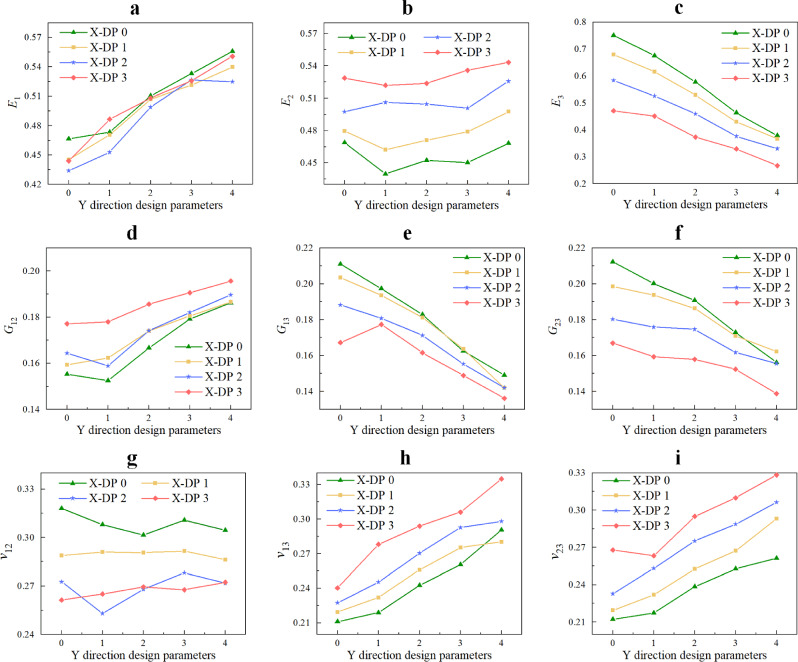
Effective engineering constants of the RVE for different $\varpi_{x}$ and $\varpi_{y}$, where X-DP *i* denotes the *x*-directional design parameter $\varpi_{x} = i,(i = 0,...,3)$.

In order to reduce the influence of other factors on the mechanical properties of RVE, the effect of pore growth randomness ($\varpi_{x} , \, \varpi_{y}$) on the mechanical properties of RVE was analyzed for a porous material model with $\alpha = 0.25$, $N = 16$, $\rho = 0.25$, $\varpi_{r} = 0$. For each pair of $\varpi_{x}$ and $\varpi_{y}$ values, i.e., $\varpi_{x}$ = 0,1,2, and $\varpi_{y}$ = 0,1,2,3,4, five porous material models were generated using five sets of independent nucleation points $p_{i0}$($i = x,y$) and random numbers $a_{i}^{\ell k} (\ell = 0,1, \ldots ,9;\;k = 1,2, \ldots ,N)$, respectively. The average values of the corresponding effective engineering constant predictions and their relative standard deviations are shown in Fig. 15 and Table 4, respectively. From Fig. 15, it can be seen that on average, as $\varpi_{y}$ increases, $E_{1}$, $G_{12}$, $v_{13}$ and $v_{23}$ increase, while $G_{13} ,G{}_{23}$ and $E_{3}$ decrease, $v_{12}$ and $E_{2}$ fluctuates slightly. The increase of $\varpi_{x}$ leads to the increase of $E_{2}$, $G_{12}$, $v_{13}$ and $v_{23}$, the decrease of $G_{13} ,G{}_{23}$ and $E_{3}$, but has little effect on $E_{1}$. In addition, as $\varpi_{x}$ increases, $v_{12}$ decreases. As $\varpi_{x}$ further increases, $v_{12}$ remains within a stable range. At the same time, for each $\varpi_{x}$ value, as $\varpi_{y}$ increases from 0 to 4, the change in the average value of $E_{1}$ is similar. For each $\varpi_{y}$ value, as $\varpi_{x}$ increases from 0 to 3, the change in the average value of $E_{2}$ is similar. This means that when $\varpi_{x}$ and $\varpi_{y}$ change, the interaction between them has little effect on the elastic modulus in the *x* and *y* directions. The effect of pore growth randomness on $E_{3}$ decreases as pore growth randomness increases. At the same time, from $\varpi_{x} = \varpi_{y} = 0$ to $\varpi_{x} = 3$,$\varpi_{y} = 4$, $E_{1}$, $E_{2}$,$G_{12}$,$v_{13}$ and $v_{23}$ increased by 18%, 16%, 26%, 58%, 55%, respectively. $E_{3}$, $G_{13} ,G{}_{23}$,$v_{12}$ decreased by 181%, 55%, 53%, 17%, respectively. This indicates that the sensitivity of the mechanical properties of RVE in the *z* direction to pore growth randomness is higher than that of the mechanical properties of RVE in the *x* and *y* directions.

**Table 4:** Relative standard deviation in effective properties for porous materials with different $\varpi_{y}$ and $\varpi_{x}$ (unit: %).

$\varpi_{x}$	$\varpi_{y}$	$E_{1}$	$E_{2}$	$E_{3}$	$G_{12}$	$G_{13}$	$G_{23}$	$v_{12}$	$v_{13}$	$v_{23}$
0	0	3.43	3.73	0.23	5.13	2.83	1.88	5.33	3.27	3.89
1	0	7.14	3.21	1.63	5.22	3.12	1.00	5.26	6.73	3.12
2	0	7.43	4.37	3.86	2.02	5.32	4.16	7.27	8.63	6.67
3	0	2.53	2.93	5.66	3.79	2.85	4.23	4.79	6.47	3.10
0	1	3.73	7.63	1.02	5.16	2.54	5.72	6.04	4.54	7.09
1	1	2.57	3.35	1.87	2.10	1.11	1.90	4.73	2.83	5.58
2	1	7.23	4.69	3.26	6.90	5.38	3.50	6.98	8.97	4.32
3	1	2.08	3.74	5.46	2.89	1.78	3.49	3.39	3.90	5.38
0	2	1.81	6.79	2.84	4.40	4.71	4.76	8.28	4.01	8.33
1	2	2.57	2.53	0.88	2.63	3.74	1.36	4.19	3.98	3.27
2	2	2.94	2.63	3.93	4.27	1.31	3.05	2.63	3.12	3.52
3	2	3.06	4.23	5.42	1.31	2.70	3.52	2.50	2.63	5.05
0	3	2.96	3.99	3.05	2.86	2.18	2.88	5.99	2.83	5.03
1	3	4.13	3.91	4.28	2.56	4.37	4.40	4.00	5.23	5.83
2	3	3.64	2.63	5.36	1.63	4.03	2.34	5.14	2.66	4.06
3	3	4.32	0.82	7.12	1.54	4.09	3.43	1.57	4.55	1.85
0	4	1.73	3.55	8.04	4.26	2.75	4.92	6.20	6.96	3.85
1	4	2.28	4.27	7.93	3.93	3.61	3.63	3.19	4.12	4.18
2	4	2.51	2.72	3.83	1.77	3.20	3.29	2.74	6.12	2.98
3	4	1.90	2.91	10.00	1.37	3.07	5.72	2.70	3.83	2.88

This is because when $\varpi_{y}$ (or $\varpi_{x}$) increases while $\varpi_{x}$ (or $\varpi_{y}$) remains unchanged, the projection area of the pores on the *XY* plane increases significantly, and the projection area of the pores on the *YZ* (or *XZ*) plane decreases as $\varpi_{y}$ (or $\varpi_{x}$) increases, but the projection area on the *XZ* (or *YZ*) plane remains unchanged. Therefore, as $\varpi_{y}$ (or $\varpi_{x}$) increases, the mechanical properties of porous materials in the *x* (or *y*) direction increase, while the mechanical properties in the *z* direction decrease. At the same time, an increase in pore growth randomness leads to an increase in the irregularity of cell wall cross-sectional area. However, from Fig. 15, it can be seen that for low porosity RVEs with a given porosity, the relative projection area of pores dominates the effect on RVE’s effective mechanical properties. In other words, as $\varpi_{x}$ or $\varpi_{y}$ increases, the randomness of pore particle distribution on the *XZ* or *YZ* plane increases, and at the same time, the randomness of pore growth in the *x* or *y* direction increases. This indicates that the mechanical properties of porous materials are affected by the randomness of pore particle growth. As pore growth randomness increases, the relative projection area of pores on the *XY* plane increases and decreases on the *XZ* and *YZ* planes; and the unevenness of cell wall cross-sectional area increases. As a result, the randomness of the growth of porous particles increases, making the mechanical properties of porous materials weaker in the *z* direction and stronger in the* x* and *y* directions.

Table 4 shows that the relative standard deviations of the effective mechanical properties predicted by the five sets of RVEs generated by different $\varpi_{y}$ and $\varpi_{x}$ are all less than 15%. This once again proves the stability of the numerical results and the repeatability of the method used in this study.

### The synergy of the three spatial design parameters

To reduce the impact of other factors on the mechanical properties of RVE, the effects of pore growth randomness ($\varpi_{x} ,\varpi_{y}$) and pore size randomness ($\varpi_{r}$) on the mechanical properties of RVE were analyzed for a porous material model with $\alpha = 0.25,N = 16$,$\rho = 0.25$ For each pair of $\varpi_{x}$, $\varpi_{y}$, and $\varpi_{r}$ values, i.e., $\varpi_{x} = 2{, }3$,$\varpi_{y} = 2{, }4$, and $\varpi_{x} = 0, \, 0.4, \, 1$, five porous material models were generated respectively. The average values of the predicted effective engineering constants and their relative standard deviations are shown in Tables 5 and 6 respectively.

**Table 5:** The average value of the effective engineering constants for different $\varpi_{r}$,$\varpi_{y}$ and $\varpi_{x}$ (unit : %).

$\varpi_{x}$	$\varpi_{y}$	$\varpi_{r}$	$E_{1}$	$E_{2}$	$E_{3}$	$G_{12}$	$G_{13}$	$G_{23}$	$v_{12}$	$v_{13}$	$v_{23}$
2	2	0	0.499	0.505	0.460	0.174	0.171	0.175	0.268	0.270	0.275
2	4	0	0.525	0.526	0.330	0.190	0.142	0.156	0.272	0.298	0.306
3	2	0	0.508	0.524	0.374	0.186	0.162	0.158	0.269	0.294	0.295
3	4	0	0.551	0.543	0.267	0.196	0.136	0.139	0.272	0.335	0.328
2	2	0.4	0.505	0.496	0.455	0.177	0.171	0.172	0.274	0.272	0.273
2	4	0.4	0.542	0.519	0.299	0.191	0.142	0.145	0.277	0.319	0.312
3	2	0.4	0.515	0.527	0.381	0.184	0.161	0.155	0.266	0.291	0.293
3	4	0.4	0.543	0.543	0.268	0.199	0.138	0.139	0.275	0.330	0.325
2	2	1	0.521	0.528	0.420	0.185	0.167	0.173	0.274	0.287	0.293
2	4	1	0.543	0.542	0.323	0.194	0.140	0.153	0.276	0.303	0.310
3	2	1	0.526	0.537	0.364	0.191	0.160	0.154	0.274	0.302	0.297
3	4	1	0.559	0.550	0.278	0.198	0.135	0.138	0.278	0.326	0.323

**Table 6:** Relative standard deviation in effective properties for porous materials with different $\varpi_{r}$,$\varpi_{y}$ and $\varpi_{x}$ (unit: %).

$\varpi_{x}$	$\varpi_{y}$	$\varpi_{r}$	$E_{1}$	$E_{2}$	$E_{3}$	$G_{12}$	$G_{13}$	$G_{23}$	$v_{12}$	$v_{13}$	$v_{23}$
2	2	0	2.94	2.63	3.93	4.27	1.31	3.05	2.63	3.12	3.52
2	4	0	2.51	2.72	3.83	1.77	3.20	3.29	2.74	6.12	2.98
3	2	0	3.06	4.23	5.42	1.31	2.70	3.52	2.50	2.63	5.05
3	4	0	1.90	2.91	10.00	1.37	3.07	5.72	2.70	3.83	2.88
2	2	0.4	4.16	4.19	4.25	1.38	4.84	1.23	5.53	4.11	3.50
2	4	0.4	3.09	3.80	4.32	1.68	2.82	4.78	3.64	2.51	2.98
3	2	0.4	5.31	4.86	5.59	4.61	4.42	5.28	5.91	3.98	6.59
3	4	0.4	1.82	2.15	8.20	1.67	3.14	1.48	3.48	4.93	4.44
2	2	1	2.68	2.94	3.08	3.15	3.37	2.35	2.97	4.23	2.81
2	4	1	3.65	2.63	12.13	3.96	5.29	7.25	3.99	7.72	1.84
3	2	1	3.13	2.91	4.49	2.65	1.88	3.11	3.34	3.20	3.86
3	4	1	1.69	2.16	9.23	2.19	6.08	5.01	2.35	2.55	3.55

As shown in Table 5, there is a superimposition effect when the three spatial design parameters work together. As $\varpi_{r}$ increases from 0 to 1, the maximum relative change rate of the average value of the effective mechanical properties of RVE corresponding to the four groups of $\varpi_{x} ,\varpi_{y}$ is 10%. When the three spatial parameters work together, the impact of $\varpi_{r}$ on the effective mechanical properties of RVE is still not significant, and the trend of the impact of spatial design parameters on the effective mechanical properties of RVE remains unchanged. In addition, the interaction of the three spatial design parameters has little effect on the equivalent mechanical impact of RVE in the *x* and *y* directions. The effective mechanical properties of RVE in the z direction decrease as the randomness of pore space increases, but when the randomness of pore space reaches a certain level, the rate of decline in the mechanical properties of RVE in the z direction slows down as the spatial design parameters continue to increase. For example: when $\varpi_{x}$ changes from 2 to 3 and $\varpi_{y}$ changes from 2 to 4, the relative changes in $E_{3}$ corresponding to three $\varpi_{r}$ values (0, 0.4, and 1) are 72%, 70%, and 51%, respectively. When $\varpi_{x} = 2$ and $\varpi_{y}$ changes from 2 to 4, the relative changes in $E_{3}$ corresponding to three $\varpi_{r}$ values (0, 0.4, and 1) are 31%, 52%, and 31%, respectively. When $\varpi_{x} = 3$ and $\varpi_{y}$ changes from 2 to 4, the relative changes in $E_{3}$ corresponding to three $\varpi_{r}$ values (0, 0.4, and 1) are 40%, 42%, and 31%, respectively.

Table 6 shows that the relative standard deviations of the effective mechanical properties of the five groups of RVEs generated by different design parameters are all less than 15%, proving the reliability and repeatability of the results.

## Experimental validation and results

### Experimental equipment and methods

To verify the reliability of the numerical method used in the previous section, the RVE with design parameter $\rho = 0.242,\;\alpha = 0.25, \, N = 16, \, \varpi_{x} = \varpi_{y} = \varpi_{r} = 0, \,$ (sample b) and the RVE with design parameter $\rho = 0.24,\;\alpha = 0.25,\; \, N = 16,\; \, \varpi_{x} = 0, \, \varpi_{y} = 1,\; \, \varpi_{r} = 0$ (sample c) were prepared using SLA 3D printing technology. The 3D printed material was photosensitive resin. The specimen’s $E_{3}$ was then determined by uniaxial compression tests. The model was then numerically solved again using Poisson’s ratio of the material supplied by the manufacturer. Finally, the values between the two were compared in order to verify the reliability of the numerical method.

**Figure 16:**
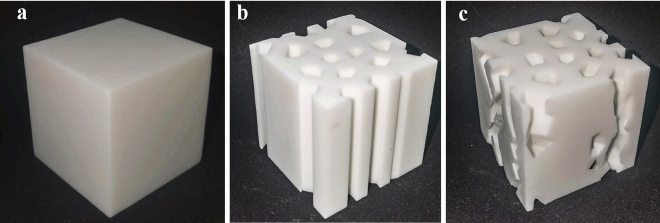
(**a**) Specimen a; (**b**) specimen b; (**c**) specimen c.

According to ASTM D1621-16 (Standard Test Method for Compressive Properties of Rigid Cellular Plastics), the sample is designed as a cube with a side length of 6 cm. Three samples are made for each structure to assure the objective and reliability of the experimental results (As shown in Fig. 16). Additionally, specimens of the same size with a porosity of 0% (labeled as specimen a) were produced for the study in order to determine the matrix material's Young's modulus. The actual porosity of the specimens is shown in Table 7. The experimental procedure was displacement-controlled, and the specimens were quasi-statically loaded using the test machine's indenter moving uniformly at a rate of 1 mm/min.

**Table 7:** The porosity of porous material specimens and their effective Young's modulus.

Specimen number	a1	a2	a3	b1	b2	b3	c1	c2	c3
Porosity (%)	0	0	0	24.13	23.95	24.49	23.90	24.01	24.06
$E_{m}$(MPa)	307.49	282.80	283.19	216.01	222.30	222.87	189.03	186.71	187.31

### Experimental results and validation

**Figure 17:**
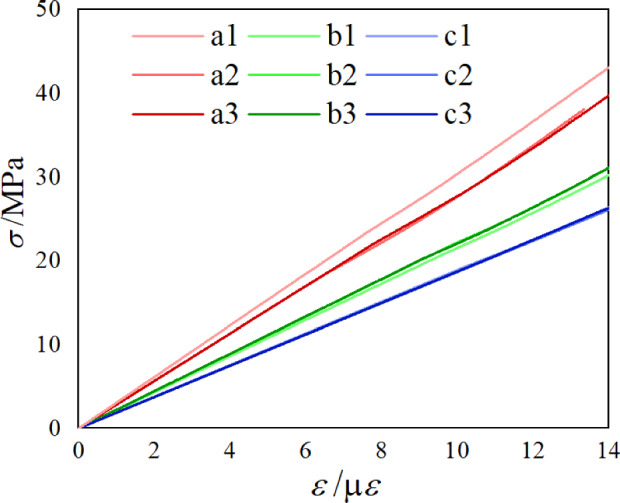
Stress–strain curve of a porous material specimen under uniaxial compression.

Stress–strain curves for each specimen under compression were generated by applying uniaxial compression in the *z* direction to several specimens, as illustrated in Fig. 17. The three curves for specimens b and c, as well as the curves for specimens a2 and a3, can be observed in the graphs and are all quite consistent, demonstrating the reliability of the experiment results. Table 7 shows the specimens' effective Young's modulus $E_{3}$ from the experiments. According to Table 7, there are significant differences between the experimental results for a1 and, a2, a3. Therefore, the mean value $\overline{E}_{3} = 282.995\;\;{\text{MPa}}$ corresponding to specimens a2, a3 and Poisson's ratio $v = 0.39$ provided by the manufacturer were taken as the material properties of the base material. Table 7 shows the effective Young's modulus $E_{3}$ that was determined by homogenizing the RVE for specimens b and c. The relative error between the numerical and experimental results was found to be less than 5% after comparing the average of the three experimental results for the same structure with the numerically computed results (as shown in Table 8).

**Table 8:** Experimental and simulation results for porous material specimens.

Specimen number	$\overline{E}_{3}$ (MPa)	FEM (MPa)	Relative error (%)
Specimen *b*	220.4	213.0	3.4
Specimen *c*	187.68	195.7	4.3

## Conclusion

For the porous solid phase of oil-bearings, a pore structure modeling approach is suggested to generate a porous material RVE with geometrical periodicity. The computational homogenization method is used to predict the RVE's effective properties. In order to investigate the relationship between the microstructure and the macroscopic mechanical properties of porous materials, the effective properties of RVEs generated from various design parameters are calculated, and the numerical results are analyzed. Finally, the availability of the numerical results was verified by performing compression tests on specimens prepared by 3D printing technology to measure the effective modulus of elasticity of the specimens. The following are the study's principal findings.

1. The predicted values of the effective mechanical properties of the RVE generated by the given design parameters are stable, proving the repeatability of the method. This means the effectiveness of the RVE generation method.
2. The 3D printing technology can be used to produce the modeling method proposed in this study. However, due to the manner in which the RVE is generated, there are localized stress concentrations in the structure that must be smoothed and other treatments applied before they can be used in industrial preparation. And for RVEs with complex structures, there are situations where the support is not easy to remove.
3. With 25% porosity, the columnar pore RVE has an elastic modulus in the *z* direction of around 0.75 $E_{s}$. At the same time, when the number of pores is high, Young's modulus in the* x* and* y* directions is close, and the shear modulus and Poisson's ratio in the *XZ* and *YZ* planes are closed.
4. For the columnar pore RVE, when the number of pores is 4, the mechanical properties of the RVE are unstable. At the same time, the irregularity of the pores has little effect on the effectiveness of the RVE.
5. The increase in the randomness of pore growth leads to a decrease in the weakening of the mechanical properties of the pores in the *x* and* y* directions, and an increase in the degree of weakening of the mechanical properties in the *z* direction. At the same time, the shear modulus in the *XY* plane increases, the shear modulus in the out-of-plane direction decreases. The Poisson’s ratio in the XY plane decreases first and then tends to stabilize as the randomness of pore growth in the x direction increases.
6. The effect of pore size randomness on the mechanical properties of RVE is similar to the effect of pore growth randomness. The difference is that the former can ignore the effect on the shear modulus in the out-of-plane direction. In addition, the effect of this factor on the effective mechanical properties of RVE is smaller than the latter.
7. The three spatial design parameters have a superimposed effect on RVE. The increase in pore space randomness (randomness of pore growth and randomness of pore size) reduces the mechanical properties of RVE in the z direction and increases the mechanical properties in the x and y directions. But the former is greater than the latter. In addition, as the randomness of pore space increases further, the rate of decline in the mechanical properties of RVE in the z direction slows down.

### Supplementary Information

Supplementary Information.

## Data Availability

All data generated or analysed during this study are included in this published article [and its supplementary information files].

## References

[1] Lin W, Klein J (2021). Recent progress in cartilage lubrication. Adv. Mater..

[2] Ruan H (2021). Effect of temperature on the friction and wear performance of porous oil-containing polyimide. Tribol. Int..

[3] Wang J, Zhao H, Huang W, Wang X (2017). Investigation of porous polyimide lubricant retainers to improve the performance of rolling bearings under conditions of starved lubrication. Wear.

[4] Yang P (2020). An ultra-simple universal model for the effective elastic properties of isotropic 3D closed-cell porous materials. Compos. Struct..

[5] Guo Z, Wang L, Guo X, Chen Y, Dong L (2020). On effective mechanical properties of two-dimensional porous materials. Int. J. Appl. Mech..

[6] Walsh JB, Brace WF, England AW (1965). Effect of porosity on compressibility of glass. J. Am. Ceram. Soc..

[7] Yao X (2013). Adaptive fluid-infused porous films with tunable transparency and wettability. Nat. Mater..

[8] Smirnov NN, Nikitin VF, Gazizova DR (2021). Evolution of a phase interface in the displacement of viscous fluids from a porous medium. Fluid Dyn..

[9] Gent AN, Thomas AG (1963). Mechanics of foamed elastic materials. Rubber Chem. Technol..

[10] Gibbson, L. J. & Ashby, M. F. Cellular solid: structure and properties. In: New York, (Cambridge Univ. Press, 1997).

[11] Li K, Gao XL, Roy AK (2003). Micromechanics model for three-dimensional open-cell foams using a tetrakaidecahedral unit cell and Castigliano's second theorem. Compos. Sci. Technol..

[12] Schmidt I (2004). Deformation induced elasto-plastic anisotropy in metal foams–modelling and simulation. Int. J. Solids Struct..

[13] Roberts AP, Garboczi EJ (2002). Elastic properties of model random three-dimensional open-cell solids. J. Mech. Phys. Solids.

[14] Pabst W, Gregorová E (2003). Derivation of the simplest exponential and power-law relations for the effective tensile modulus of porous ceramics via functional equations. J. Mater. Sci. Lett..

[15] Pabst W, Gregorová E (2004). Mooney-type relation for the porosity dependence of the effective tensile modulus of ceramics. J. Mater. Sci..

[16] Guo Z (2017). A universal model for predicting the effective shear modulus of two-dimensional porous materials. Mech. Mater..

[17] Mori T, Tanaka K (1973). Average stress in matrix and average elastic energy of materials with misfitting inclusions. Acta Metall..

[18] Christensen RM, Lo KH (1979). Solutions for effective shear properties in three phase sphere and cylinder models. J. Mech. Phys. Solids.

[19] Silva MJ, Hayes WC, Gibson LJ (1995). The effects of non-periodic microstructure on the elastic properties of two-dimensional cellular solids. Int. J. Mech. Sci..

[20] Silva MJ, Gibson LJ (1997). The effects of non-periodic microstructure and defects on the compressive strength of two-dimensional cellular solids. Int. J. Mech. Sci..

[21] Chen C, Lu TJ, Fleck NA (1999). Effect of imperfections on the yielding of two-dimensional foams. J. Mech. Phys. Solids.

[22] Li K, Gao XL, Subhash G (2005). Effects of cell shape and cell wall thickness variations on the elastic properties of two-dimensional cellular solids. Int. J. Solids Struct..

[23] Gan YX, Chen C, Shen YP (2005). Three-dimensional modeling of the mechanical property of linearly elastic open cell foams. Int. J. Solids Struct..

[24] Li K, Gao XL, Subhash G (2006). Effects of cell shape and strut cross-sectional area variations on the elastic properties of three-dimensional open-cell foams. J. Mech. Phys. Solids.

[25] Li B, Wang B, Reid SR (2010). Effective elastic properties of randomly distributed void models for porous materials. Int. J. Mech. Sci..

[26] Tarantino M, Zerhouni O, Danas K (2019). Random 3D-printed isotropic composites with high volume fraction of pore-like polydisperse inclusions and near-optimal elastic stiffness. Acta Mater..

[27] Anoukou K, Brenner R, Hong F, Pellerin M, Danas K (2018). Random distribution of polydisperse ellipsoidal inclusions and homogenization estimates for porous elastic materials. Comput. Struct..

[28] Zerhouni O, Tarantino M, Danas K (2019). Numerically-aided 3D printed random isotropic porous materials approaching the Hashin-Shtrikman bounds. Compos. Part B: Eng..

[29] Lopez-Pamies O, Goudarzi T, Danas K (2013). The nonlinear elastic response of suspensions of rigid inclusions in rubber: II—a simple explicit approximation for finite-concentration suspensions. J. Mech. Phys. Solids.

[30] Segurado J, Llorca J (2002). A numerical approximation to the elastic properties of sphere-reinforced composites. J. Mech. Phys. Solids.

[31] Xu X (2021). Effect of preparing conditions on gas permeability parameters of porous SiC ceramics. J. Eur. Ceram. Soc..

[32] Gitman IM, Askes H, Sluys LJ (2007). Representative volume: Existence and size determination. Eng. Fract. Mech..

[33] Datoo MH (2012). Mechanics of Fibrous Composites.

[34] Hill R (1972). On constitutive macro-variables for heterogeneous solids at finite strain. Proc. R. Soc. Lond. A Math. Phys. Sci..

[35] Mondal DP, Ramakrishnan N, Suresh KS, Das S (2007). On the moduli of closed-cell aluminum foam. Scr. Mater..

[36] Babu KP, Mohite PM, Upadhyay CS (2018). Development of an RVE and its stiffness predictions based on mathematical homogenization theory for short fibre composites. Int. J. Solids Struct..

