# Supplementary material for: Establishment of the microstructure of porous materials and its relationship with effective mechanical properties
Source: Sci Rep. 2023 Oct 23;13:18064. doi: 10.1038/s41598-023-43439-6 (PMC10593753; doi:10.1038/s41598-023-43439-6)
Supplement: Supplementary file 1 — Supplementary Information. [file 41598_2023_43439_MOESM1_ESM.docx]

Highlights

A new modeling method is proposed for the solid phase of porous liquid storage media

From the perspective of pore distribution, the effect of microstructure of porous materials on their effective mechanical properties was studied

The effective engineering constants of the porous material model are predicted using the computational homogenization method
